# hERG channel agonist NS1643 strongly inhibits invasive astrocytoma cell line SMA-560

**DOI:** 10.1371/journal.pone.0309438

**Published:** 2024-09-06

**Authors:** Kieran W. Benn, Patrick H. Yuan, Harvey K. Chong, Stanley S. Stylii, Rodney B. Luwor, Christopher R. French

**Affiliations:** 1 Neural Dynamics Laboratory, Department of Medicine, University of Melbourne, Melbourne, Victoria, Australia; 2 Department of Surgery, The Royal Melbourne Hospital, The University of Melbourne, Melbourne, Victoria, Australia; 3 Department of Neurosurgery, Royal Melbourne Hospital, The University of Melbourne, Victoria, Australia; 4 Department of Medicine, Royal Melbourne Hospital, The University of Melbourne, Melbourne, Victoria, Australia; Bowen University, NIGERIA

## Abstract

Gliomas are highly malignant brain tumours that remain refractory to treatment. Treatment is typically surgical intervention followed by concomitant temozolomide and radiotherapy; however patient prognosis remains poor. Voltage gated ion channels have emerged as novel targets in cancer therapy and inhibition of a potassium selective subtype (hERG, Kv11.1) has demonstrated antitumour activity. Unfortunately blockade of hERG has been limited by cardiotoxicity, however hERG channel agonists have produced similar chemotherapeutic benefit without significant side effects. In this study, electrophysiological recordings suggest the presence of hERG channels in the anaplastic astrocytoma cell line SMA-560, and treatment with the hERG channel agonist NS1643, resulted in a significant reduction in the proliferation of SMA-560 cells. In addition, NS1643 treatment also resulted in a reduction of the secretion of matrix metalloproteinase-9 and SMA-560 cell migration. When combined with temozolomide, an additive impact was observed, suggesting that NS1643 may be a suitable adjuvant to temozolomide and limit the invasiveness of glioma.

## Introduction

Gliomas are the most common primary central nervous system tumour and one of the most deadly [1]. Advancement in the standard of care has failed to translate into improved prognosis for glioma patients, with five-year survival improving only 1% over the last three decades for glioblastoma (GBM) [2, 3]. The current standard of care for the treatment of brain cancer involves maximal resection of the tumour, followed by radiotherapy and concomitant temozolomide (TMZ), known as the Stupp protocol [4, 5]. This treatment protocol extends median survival to only 15 months for GBM patients underpinning the need for new therapeutics to improve both prognosis and quality of life amongst this population [6].

There is increasing evidence that ion channels play a significant role in tumorigenesis and cancer progression [7, 8]. The human Ether-à-go-go Related Gene (hERG), also known as KCNH2, encodes for a component of Kv11.1, a voltage-gated potassium channel involved in homeostatic regulation, as well as rhythmogenesis in cardiac and neural tissue [9–13]. Overexpression of hERG channels is implicated in driving both malignant transformation and tumour progression [14–20]. It has also been demonstrated to be associated with neo-angiogenesis, upregulation of cell proliferation, and increases in cell motility and invasion [12, 21]. Importantly, the overexpression of hERG channels is a marker for poor patient prognosis [15, 18, 22]. In GBM patients, high hERG expression resulted in poorer overall survival; 43.5 weeks compared to 60.9 weeks in patients with low hERG expression [23]. Notably, this difference in survival was reduced when GBM patients with hERG overexpression were incidentally treated with non-torsadogenic hERG channel blockers, demonstrating potential antitumor efficacy through the modulation of hERG channels. Expression of KCNH2 is further variable by tumour histological subtype and may be examined through microarray data from gene expression database Oncomine [24].

Through inhibition of hERG channel function, tumour cells with high hERG expression can undergo apoptosis and show a decline in their rate of proliferation [14, 25–28]. However, therapies targeting hERG channel function are limited due to the cardiac risk profile of hERG channel antagonists, posed through drug-induced arrhythmias and sudden cardiac death [29]. Interestingly, the narrow range of function of hERG channels enables hyperstimulation *via* hERG channel agonists as a viable method to irreversibly inhibit the proliferation of tumour cells that are ectopically expressing the channel, while simultaneously minimising cardiotoxic risk [30, 31].

The hERG channel agonist NS1643 has been reported to reduce cell proliferation in rodent and human glioma cell lines [32]. Herein, the SMA-560 mouse glioma cell line was treated with the hERG channel agonist NS1643 and in combination with TMZ, resulting in a strong inhibition of cell proliferation. Our data supports further research into the potential use of N1643 in the clinical management of glioma patients.

## Materials and methods

### SMA-560 cell culture

The SMA-560 tumour cell line, which was generously provided by Dr. Hui Lau (Royal Melbourne Hospital), was cultured in DMEM (10313–021, Gibco) supplemented with (in v/v) 10% FBS (12003C, SAFC Biosciences, Australia), 1% penicillin-streptomycin (15140122, Gibco) and 1% GlutaMax (35050061, Gibco) and incubated at 37°C with 5% CO_2_ in a humidified environment [33].

### Cell proliferation assays

Cells were seeded in 96-well plates and allowed to adhere overnight. Triplicate wells were treated with varying concentrations of NS1643 as indicated for 72 h. Cells were subsequently lysed and cell viability determined relative to the vehicle control using a commercially available Cell Titer-Glo kit (Promega) following manufacturer’s instructions.

### Scratch assay

Cells were seeded into 6-well plates and allowed to adhere for 48 h. A 1 mL pipet tip was used to create the scratch. NS1643 (Sigma Aldrich; CAS 448895-37-2) or temozolomide (Xi’an D-Sung Health Biotechnology; CAS 85622-93-1) were prepared in the cell medium at a stock concentration of 20 mM and 100 mM, respectively.

Cell migration was monitored using an IX50 inverted system microscope (Olympus) and DFC3000 G microscope camera (Leica) controlled by Leica Application Suite Version 4.6. Images were taken with a 4X objective at 0, 24 and 48 h post-scratch and ImageJ (Schneider et al., 2012; version 1.53t) was used to analyse the closure of the scratch to determine the cell migration rate.

### Gelatinase zymography

Cells were seeded in six-well plates (Corning) and were allowed to adhere overnight before washing with sterile PBS and subsequent incubation in serum-free OptiMEM (Thermofisher Scientific) for 24 h. One-hundred-microliter aliquots of the conditioned OptiMEM medium was then sampled and centrifuged at 1000×*g* (4°C) for 10 min. Gelatin-based zymography was performed with the conditioned OptiMEM media samples using 10% gelatin-substrate zymography NuPAGE precast gels (Invitrogen, Australia) at 125 V for 1.5 h and the gels were stained with Simply-Blue Stain (Life Technologies), followed by washing in distilled water until clear gelatinolytic bands were visible. The gels were scanned using a flatbed scanner for further densitometric analysis using Image J (version 1.51f).

### Identification of hERG channels *via* patch clamp

Healthy SMA-560 cells were selected for recordings. Fire polished borosilicate micropipettes (1.5–2.5 MΩ) were fabricated with a Sutter Instrument model P-1000 micropipette puller and filled with a KF-based intracellular solution (in mM: 130 KF, 1 CaCl_2_, 1 MgCl_2_, 5 NaCl, 10 HEPES, 10 phosphocreatine, 4 MgATP, 0.3 GTP, 5 EGTA) (pH 7.25) and whole cell voltage clamp recordings were carried out in a temperature controlled room at 22°C in a high K^+^ external solution (in mM: 150 KCl, 4 NaCl, 10 HEPES, 2 MgCl_2_, 2 CaCl_2_). Currents were recorded with an AM systems model 2400 patch clamp amplifier with a sampling interval of 10μs and Clampfit 10 software with a Digidata 1322A interface. The inhibitor-sensitive currents were obtained by subtracting the recorded currents in the presence of a hERG-channel inhibitor E4031 from those recorded in the absence of the drug.

At the start of experimentation on each neuron, the high K^+^ external solution was perfused into the recording chamber. From a holding potential of -100 mV the cell was depolarised to +40 mV for 500 ms followed by step changes in voltage with 500 ms negative repolarising pulses from -80 mV to 170 mV with a 10 mV decrement at each sweep. Cells were perfused with hERG channel inhibitor E4031 in high K^+^ external solution and the hERG protocol repeated to obtain current tracing following treatment with E4031. E4031 was washed out at a holding voltage of -100 mV, and the hERG protocol repeated to obtain current tracing following washout. Analysis of current tracing was performed using Clampfit 10 software.

### Oncomine data mining

Oncomine was used to compare expression levels of KCNH2 in different histological grades of glioma relative to normal brain tissue from different studies [24]. Search criteria examined primary articles only from the previous 10 years, relevant to KCNH2 expression in human gliomas.

## Results

### NS1643 inhibits cell proliferation

**Fig 1:**
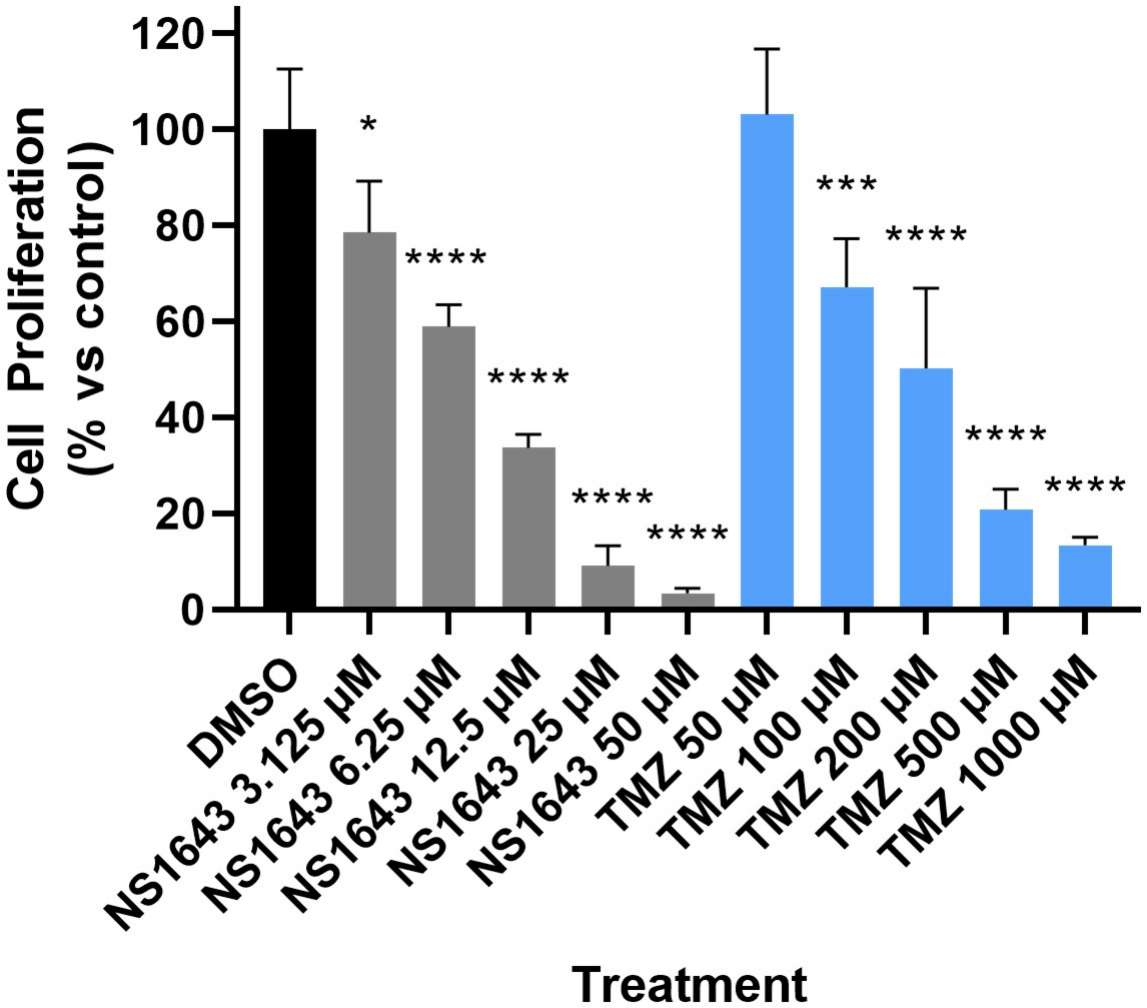
Cell proliferation assays: NS1643 inhibits cell proliferation. Cells were treated with indicated concentrations of NS1643 (n = 5) or TMZ (n = 5) for 72 h. Cell proliferation determined *via* Cell Titer-Glo kit. All statistical analyses were performed using GraphPad Prism 10. Data was analysed using repeated measures one-way ANOVA followed by a Dunnett’s test relative to DMSO control cells. Values were determined to be significant if p<0.05 (*), p<0.01 (**), p<0.001, and p<0.0001 (***).

When SMA-560 cells were treated with NS1643 at concentrations greater than or equal to 3.125 μM over a 72 h period a statistically significant reduction in cell proliferation was observed, relative to the control (Fig 1). Notably, when treated with NS1643 concentrations greater than 25 μM, cell proliferation was almost arrested; 3.385%. ± 1.034 for 50 μM NS1643. Comparatively, high dose temozolomide treatment at 1000 μM resulted in 13.4353% ± 1.658 cell proliferation, relative to the control.

### NS1643 reduces matrix metalloproteinase-9 secretion and migration in SMA-560 cells

**Fig 2:**
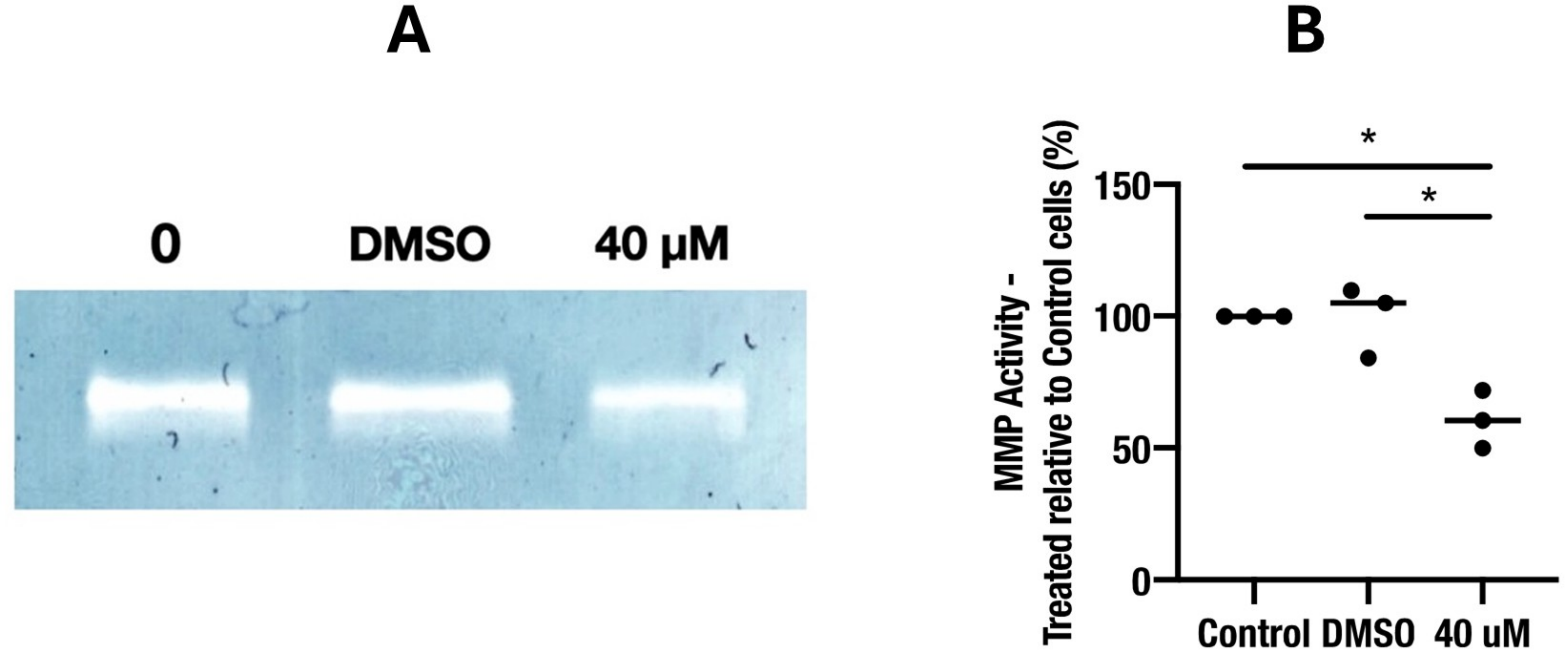
NS1643 (40 μM) reduces MMP-9 secretion in SMA-560 cells: A) Gelatinase zymogram analysis of serum-reduced Optimem conditioned medium showing MMP-9 secretion SMA-560 cells (0 μM NS1643, DMSO control, 40 μM NS1643). B) Densitometric analysis of 3 zymogram experiments. All statistical analyses were performed using GraphPad Prism 10. Data was analysed using repeated measures one-way ANOVA followed by a Tuckey’s post hoc test. Values were determined to be significant if p<0.05 (*), p<0.01 (**), p<0.001, and p<0.0001 (***).

Treatment of SMA-560 cells with a 40 μM concentration of NS1643 significantly reduces matrix metalloproteinase-9 (MMP) secretion (37.67% ± 6.49) (Fig 2).

**Fig 3:**
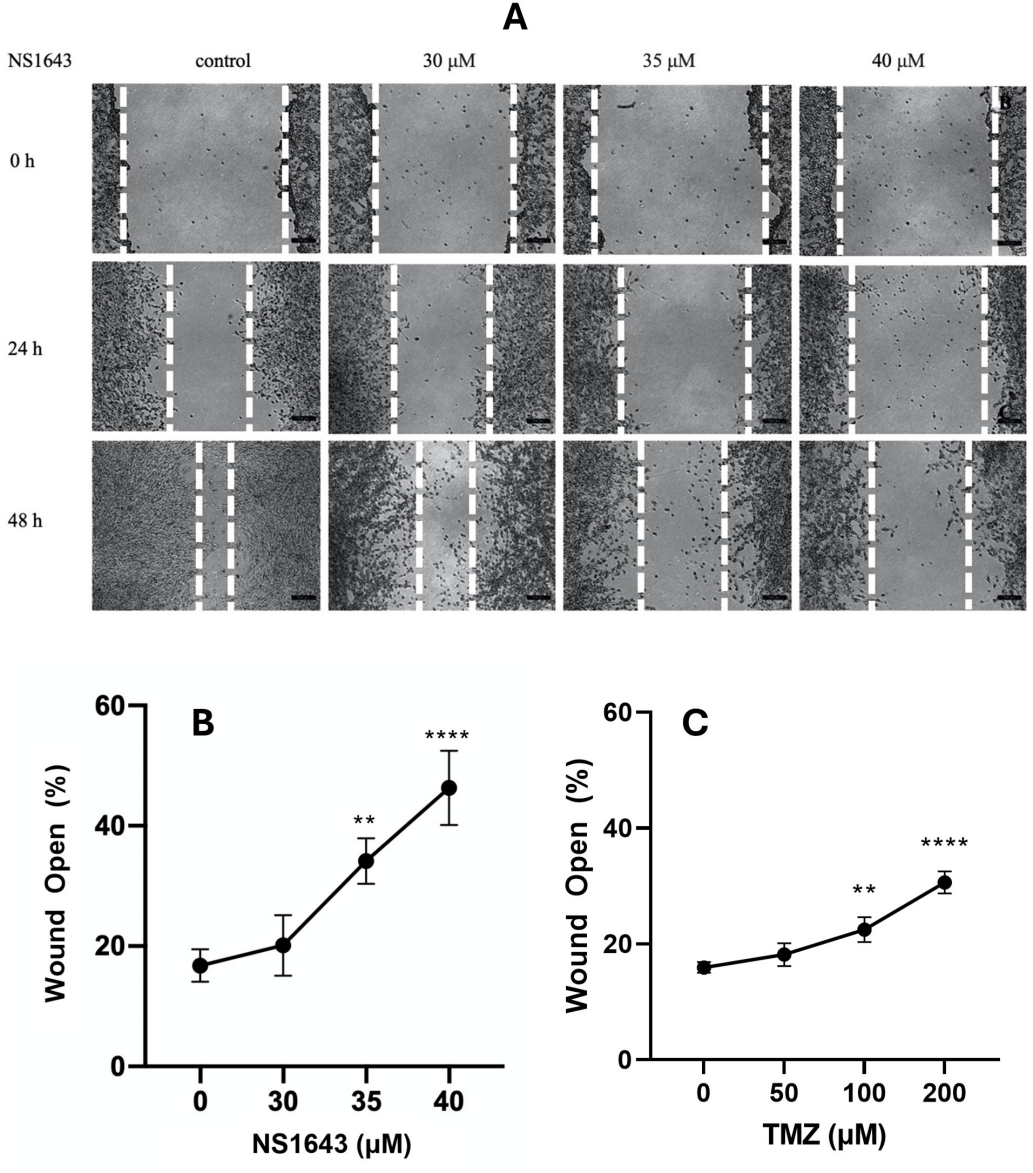
Scratch assays of SMA-560 cells following 48 h treatment with NS1643 or TMZ: A) Images of SMA-560 scratch assay at varying timepoints following NS1643 treatment (n = 4; Dotted lines mark the borders of the wound area). A,B) Quantification of scratch assays with pixel analysis demonstrating % of wound area remaining open at 48 h following treatment with either NS1643 or TMZ at indicated concentration. Data was analysed using repeated measures one-way ANOVA followed by a Dunnett’s post hoc test relative to DMSO control cells. Values were determined to be significant if p<0.05 (*), p<0.01 (**), p<0.001 (***), and p<0.0001 (****).

NS1643 was effective in reducing wound healing of SMA-560 cells relative to the untreated control (Fig 3). When treated with 40 μM of NS1643, SMA-560 exhibit statistically significant reduction in wound healing capacity resulting in an average remaining wound area at 48 h of 46.30% ± 3.08 (mean ± SEM). Compared to untreated control cells, which displays 16.78% ± 1.34 (mean ± SEM) of the original wound area, this amounts to a 29.53% difference in wound area. Treatment with 200 μM TMZ resulted in 31.05% ± 1.267 (mean ± SEM) wound area remaining, representative of a 15.25% difference in wound area when compared to 40 μM NS1643.

### NS1643 and temozolomide combination treatment displays additivity in SMA-560 cells

**Fig 4:**
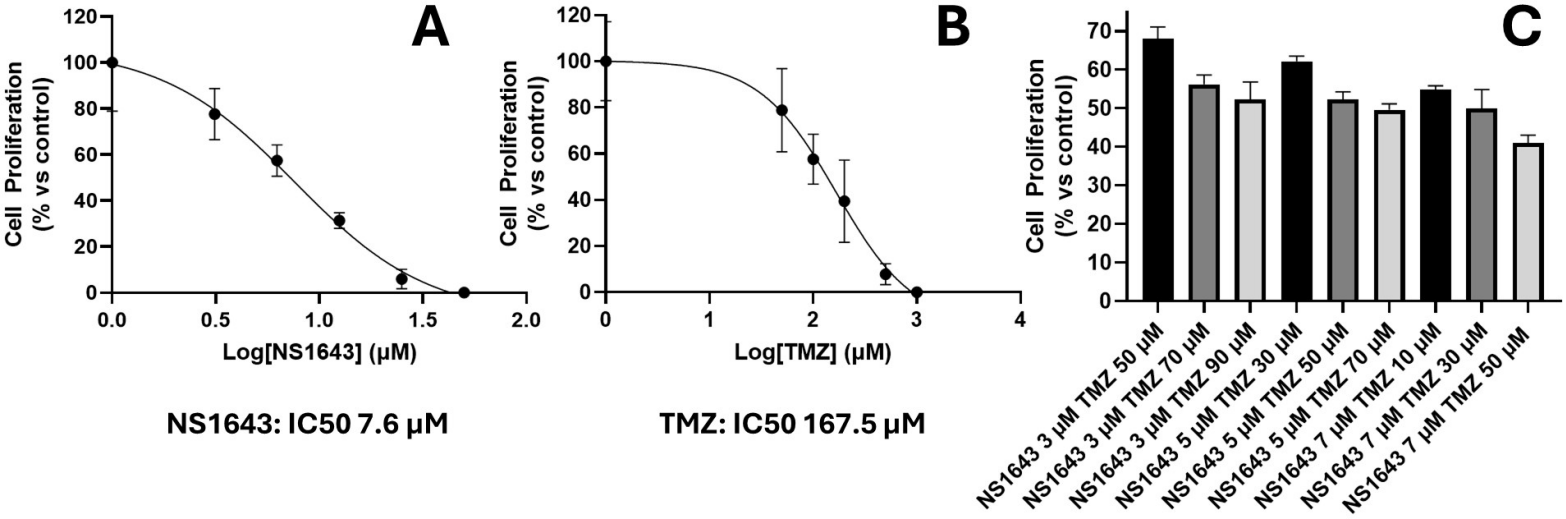
NS1643 and TMZ display simple additivity: A) IC_50_ of NS1643 for SMA-560 as determined by cell proliferation assay. B) IC_50_ of temozolomide for SMA-560 cells as determined by cell proliferation assay. C) Cell proliferation following combination treatment of SMA-560 cells with NS1643 and TMZ at indicated concentrations. Cell proliferation determined *via* Cell Titre Glo kit following 72h treatment.

The IC_50_ values for NS1643 and TMZ for cell proliferation in the SMA-560 cells were determined to be 7.6 μM and 167.5 μM, respectively (Fig 4). Following this, a range of paired concentrations below for IC_50_ for both NS1643 and TMZ were assessed to determine a possible synergistic relationship as previously described [34]. Superadditivity indicative of synergism was not observed, however low dose combination treatment produced a substantial reduction in cell proliferation, relative to DMSO control, suggestive of additivity (Fig 4)

### hERG channel inhibitor E4031 reduces hERG channel characteristic tail current amplitude in SMA-560 cells

**Fig 5:**
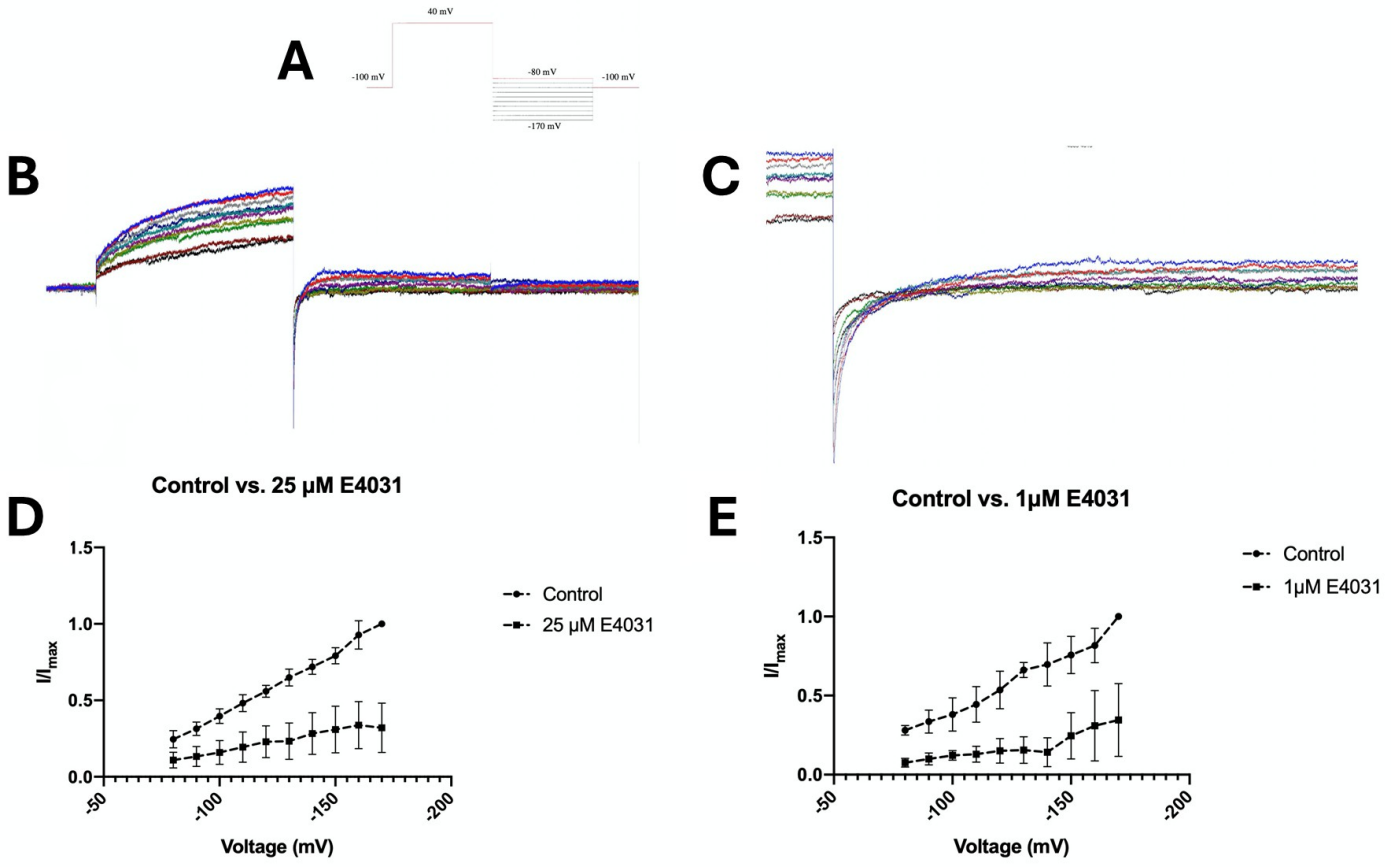
Characterisation of E-4031-senstive currents in the SMA-560 cells (n = 5): A) hERG protocol; cells were held at -100 mV prior to depolarisation the cell at +40 mV for 500 ms and stepping down to a series of 500 ms repolarising pulses from -80 mV to -170 mV, with a 10 mV decrement each sweep. B) Whole current tracing of hERG channel inhibitor E-4031-subtracted (25μM) currents elicited by a hERG channel-specific voltage protocol. C) Enlarged representative E-4031-substracted tail currents. D, E) Normalised tail current amplitude elicited by repolarising currents. Peak tail currents were measured and normalised to the maximal tail current produced at -170 mV. Amplitude is compared.

Patch clamp investigation of healthy SMA-560 cells, when subject to the hERG protocol and dosed with E-4031, at concentrations of 25 μM or 1 μM, revealed clear inhibition compatible with hERG currents as previously described [35]. Current subtraction between the currents recorded in the presence and the absence of the E4031 was carried out to obtain the E-4031-sensitive currents (Fig 5). A steady outward component, as well as a hook shaped tail current was observed; highly characteristic of hERG channels.

### Gene KCNH2 is expressed differentially independent of brain tumour histological subtype

Microarray data from publicly available gene expression database *Oncomine* was extracted to examine the expression level of KCNH2 in different histological subtypes of human brain cancers, relative to normal brain tissue (Table 1) [24]. Independent studies showed a significant difference in the value of gene expression (*p* ≤ 0.05) in human brain cancers compared with normal human brain tissues [36–39].

**Table 1:** Analysis of KCNH2 mRNA levels in human brain cancer tissues.

Study	Beroukhim [36] [187]				French [37] [101]		Bredel [38] [54]		Murat [39] [84]
**Percentile**	97	94	92	90	91	85	87	85	78
**Tumour Type**	GBM	GBM*	AA	AO	AOA	AO	AOA	AO	GBM
**Percentage Change (%)**	117.5	113.9	109.5	112.6	185.2	171.9	281.5	296.9	125.2
**P-value**	3.49E-13	5.88E-4	0.023	0.038	0.005	8.98E-4	0.027	0.031	0.005

Percentile–percentile of overexpression for KCNH2 mRNA. GBM–glioblastoma; AA–anaplastic astrocytoma; AO–anaplastic oligodendroglioma; AOA–anaplastic oligoastrocytoma.

*—Secondary GBM

**Fig 6:**
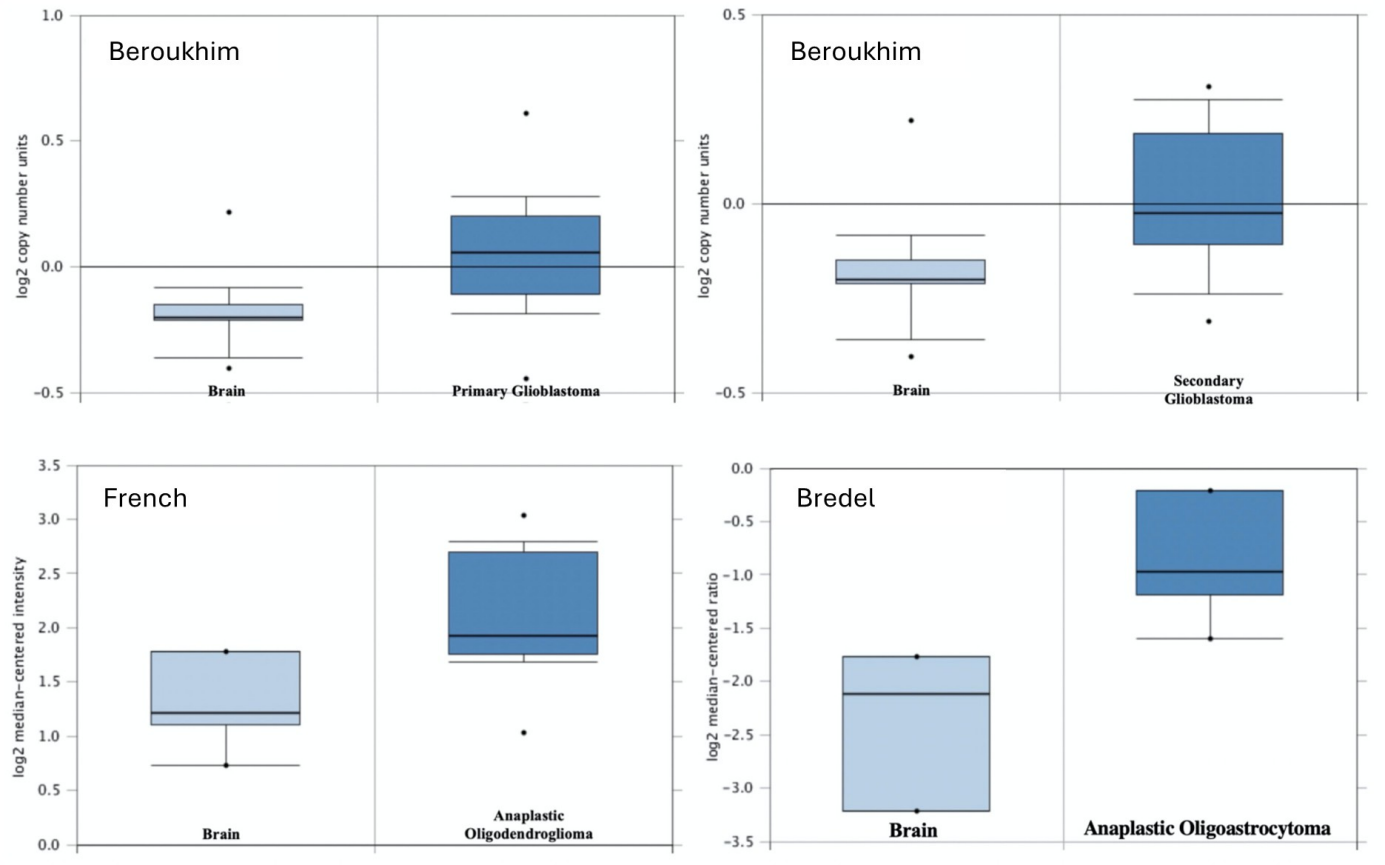
Box plot indicating KCNH2 expression level in different studies: Oncomine dataset; threshold by P-Value: 0.05; Fold Change: 1; Gene rank: Top 20%.

In the Beroukhim study, KCNH2 was significantly overexpressed in primary and secondary glioblastoma compared to normal brain and was among the top 3% and 6% overexpressed genes, respectively (Table 1). The study also showed that KCNH2 was significantly overexpressed in anaplastic astrocytoma and anaplastic oligodendroglioma (Fig 6) [36] Similarly, in the study conducted by French, KCNH2 was also found to be significantly overexpressed and was ranked among the top 9–15% overexpressed genes in anaplastic oligoastrocytoma and anaplastic oligodendroglioma [37]. A study by Murat revealed that KCNH2 was overexpressed in GBM and was ranked among the top 22% of overexpressed genes [39].

## Discussion

Malignant gliomas are among the deadliest cancers and represent a disproportionately high percentage of all cancer-related deaths annually, despite their low prevalence [40]. The five-year relative survival of GBM patients has only improved by 1% over the last three decades, signifying that despite some appreciable advances in the treatment of gliomas, a dearth of efficacious therapeutics that can effectively improve the prognosis for glioma patients still remains [2].

Increasing evidence suggests that potassium channels play many fundamental roles in the pathogenesis of glioma [41]. Overexpression or disruption of ion channel function can promote cell proliferation and cell migration [42–44]. The voltage-gated potassium channel, hERG, has been shown to be involved in the upregulation of cell proliferation, cell motility, cell invasion as well as in the process of angiogenesis [12, 21]. Treatment with hERG channel antagonists has been shown to increase apoptosis and reduce proliferation in melanoma, non-small cell lung cancer, and GBM [14, 25, 26]. However, the therapeutic action of hERG channel blockade is limited by severe cardiotoxicity [45]. Off-target blockade of hERG channels is known to produce drug-induced long QT syndrome, which increases the risk of cardiac arrhythmias, namely *torsade de pointes*, and sudden cardiac death [46]. Due to the cardiac risk profile, several hERG channel antagonists have been discontinued [13].

Further research has demonstrated that hERG channels have a narrow range of function during cell cycle progression [47]. Consequently, both constitutive inhibition and hyperstimulation of hERG channels have been proposed to be capable of inducing senescence [30]. This has presented an alternative route to extract chemotherapeutic utility from hERG channel modulation and significant cardiotoxicity has yet to observed in animal models [31]. Treatment of breast cancer cells expressing hERG channels with NS1643, irreversibly inhibited proliferation [30]. Although they were not able to detect an apoptotic event, it was however observed that NS1643 treated cells were preferentially arrested in the G0/G1 phase with increased expression of the tumour suppressor proteins p21 and p16. Similar results are seen in this study where treatment of SMA-560 cells with NS1643 results in potently decreased cell proliferation. Notably, at concentrations of NS1643 greater than 25 μM, cell proliferation of SMA-560 cells was reduced to approximately 9% of the control.

The mechanism of inhibition is purported to be through the downregulation of cyclin E2 [48, 49]. Cyclin E2 plays a critical role in cell cycle phase G1 progression and is described as rate limiting for S phase entry [49]. Treatment with NS1643 has been demonstrated to reduce levels of cyclin E2, possibly through increased rates of degradation through the ubiquitin-proteasome pathway [49]. It also has been reported that down-regulation of cell proliferation may occur *via* a calcineurin-dependent transcription of p21^waf/cip^ induced by treatment with NS1643 [50]. Notably, an *in vivo* model of breast cancer demonstrated that mice treated with NS1643 did not exhibit significant cardiac dysfunction, affirming a reduced cardiac risk profile of hERG channel hyper-stimulation, comparatively to blockade [31]. Treatment with NS1643 did produce a small elevation in the heart rate of some mice, however, this can be appropriately managed with routine pharmacotherapy. Treatment of SMA-560 cells with NS1643 at 50 μM, also resulted in a more significant decrease in cell proliferation compared to high dose treatment with TMZ at 1000 μM (Fig 1). Notably, the observed sensitivity of SMA-560 cells to temozolomide appears to be significantly greater than previously reported, perhaps due to differing methods of viability assessment [51]. The absence of reported cardiotoxicity in animal models, and potent anti-tumour activity, suggests treatment with NS1643 may offer therapeutic utility for the management of glioma.

Treatment of glioma is intrinsically challenging due to the inherent limitations of surgical intervention only providing a maximal safe resection of the tumour and often leaving residual tumour tissue [52]. Furthermore, glioma cells surviving subsequent chemotherapy and radiotherapy see a shift towards a more invasive phenotype through increased MMP activity [53–56]. Invasion is facilitated by cell membrane bound, dynamic, actin-rich structures known as invadopodia that secrete MMP-2 and MMP-9 [56]. MMP secretion results in degradation of the surrounding extracellular matrix, thereby facilitating migration, invasion, and metastasis [57]. Adjuvant therapies for glioma should aim to kill cells surviving the Stupp protocol, as well as inhibit the invasive capability of glioma. Treatment of SMA-560 cells with 40 μM NS1643 reduced MMP-9 secretion over a 24 h period suggesting NS1643 may reduce the invasivity of glioma. Furthermore, reduced wound healing, and migratory capacity, of SMA-560 cells was observed following treatment with NS1643.

The diverse microenvironment of gliomas is driving current research toward combinatorial therapy [58]. TMZ alkylates purine bases within DNA generating a persistent methyl adduct in the template strand [59]. This adduct is clastogenic, leading to collapse of the replication fork and cell cycle arrest. Senescence follows and concomitant action of downstream apoptotic proteins results in cell death. This mechanism of action is distinct from NS1643, suggesting combinatorial therapy may be a viable treatment strategy [30, 31]. Combination treatment of SMA-560 cells with NS1643 and TMZ displayed simple additivity as determined by cell proliferation assay, suggesting NS1643 may be a suitable adjuvant.

Bioinformatics data, obtained from Oncomine, indicates that KCNH2 gene is expressed differentially independent of brain cancer histological subtypes [36–39]. The degree of overexpression is most prominent in diffuse astrocytic and oligodendroglial tumours. Herein, patch-clamp investigation of SMA-560 cells is highly suggestive of hERG channel expression. The hERG channel shares structural similarities with the depolarisation-activated potassium channel family, however displays inward rectification expected of potassium channels with two transmembrane segments [60]. Upon repolarisation to a negative membrane potential, channel conductance increases dramatically to a large value before returning slowly to the resting closed state; this produces a prominent tail characteristic of hERG channels. The underlying mechanism for the gating kinetics of hERG channels is proposed to be the result of an inactivation mechanism that deactivates channels at positive potentials, but quickly recovers at negative potentials [61–63]. SMA-560 cells were treated with hERG channel antagonist E-4031 to visualise underlying hERG channel kinetics. Treatment with the inhibitor produced currents with hook-like characteristics consistent with classical hERG channel blockade [62, 64]. Amplitude of tail currents was proportional to increasingly negative potential, suggesting a dependence on repolarising potential. This is due to rapid recovery of hERG channels from inactivation to an open state, before slowly returning to the deactivated state [64]. SMA-560 cells are a well-established glioma model, representative of differentiated anaplastic astrocytoma and the patch-clamp data provides constitutive evidence for the presence of hERG channels [65]. This suggests a direct mechanism of action for NS1643 on SMA-560 cells, instead of non-specific toxicity.

## Conclusion

In summary, the presence of hERG channels is recognised in SMA-560 cells through patch clamp investigation and hERG channel agonist NS1643 is demonstrated to almost entirely halt proliferation of anaplastic astrocytoma cell line SMA-560 at concentrations greater than 25 μM. The invasive capability of SMA-560 would also be impacted through the reduced secretion of MMP-9. In addition, the migratory ability of the SMA-560 cell line appears to be affected due to the observed decrease in wound closure. Furthermore, NS1643 displays additivity for combination treatment with TMZ, suggesting NS1643 may be a useful adjuvant to the current standard of care. Future work should aim to expand these findings to human cell lines and *in vivo* study.

## Supporting information

S1 Fig Gelatinase zymogram measuring MMP-9 secretion following NS1643 treatment: Image taken 24 h after treatment with 40 μM NS1643. (TIF)
