# Correction: hERG channel agonist NS1643 strongly inhibits invasive astrocytoma cell line SMA-560

**DOI:** 10.1371/journal.pone.0316845

**Published:** 2024-12-30

**Authors:** Kieran W. Benn, Patrick H. Yuan, Harvey K. Chong, Stanley S. Stylli, Rodney B. Luwor, Christopher R. French

The fourth author’s name is spelled incorrectly. The correct name is: Stanley S. Stylli. The correct citation is: Benn KW, Yuan PH, Chong HK, Stylli SS, Luwor RB, French CR (2024) hERG channel agonist NS1643 strongly inhibits invasive astrocytoma cell line SMA-560. PLOS ONE 19(9): e0309438. https://doi.org/10.1371/journal.pone.0309438

## References

[1] Benn KW, Yuan PH, Chong HK, Stylii SS, Luwor RB, French CR (2024) hERG channel agonist NS1643 strongly inhibits invasive astrocytoma cell line SMA-560. PLOS ONE 19(9): e0309438. doi: 10.1371/journal.pone.0309438 39240809 PMC11379238

