# Supplementary figures and images for: hERG channel agonist NS1643 strongly inhibits invasive astrocytoma cell line SMA-560
Source: PLoS One. 2024 Sep 6;19(9):e0309438. doi: 10.1371/journal.pone.0309438 (PMC11379238; doi:10.1371/journal.pone.0309438)

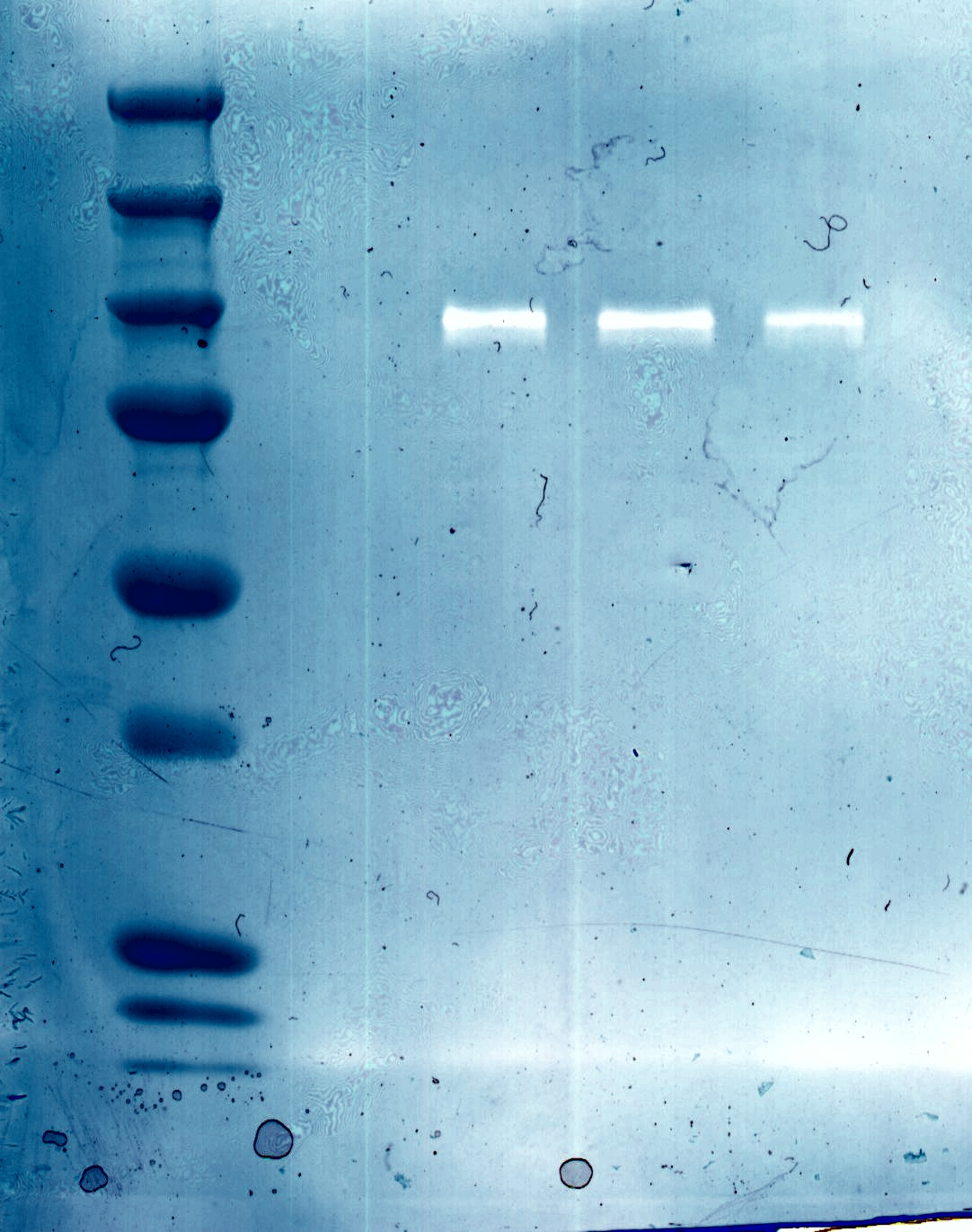

Supplement: S1 Fig — (TIF) [file pone.0309438.s001.tif]
